# Predictive factors and prognosis of immune checkpoint inhibitor-related pneumonitis in non-small cell lung cancer patients

**DOI:** 10.3389/fonc.2023.1145143

**Published:** 2023-04-26

**Authors:** Xiaoyu Liu, Na Hao, Shuangning Yang, Jieyao Li, Liping Wang

**Affiliations:** ^1^ Department of Oncology, The First Affiliated Hospital of Zhengzhou University, Zhengzhou, China; ^2^ Department of Oncology, The First Affiliated Hospital of Wannan Medical College, Wuhu, China

**Keywords:** lung cancer, immune checkpoint inhibitor-related pneumonitis, hemoglobin, albumin, survival

## Abstract

**Objective:**

To investigate the influencing factors and prognosis of immune checkpoint inhibitor-related pneumonitis (CIP) in advanced non-small cell lung cancer (NSCLC) patients during or after receiving immune checkpoint inhibitors(ICIs).

**Methods:**

The clinical and laboratory indicator data of 222 advanced NSCLC patients treated with PD-1/PD-L1 inhibitors at the First Affiliated Hospital of Zhengzhou University between December 2017 and November 2021 were collected retrospectively. The patients were divided into a CIP group (n=41) and a non-CIP group (n=181) according to whether they developed CIP or not before the end of follow-up. Logistic regression was used to evaluate risk factors of CIP, and Kaplan‒Meier curves were used to describe the overall survival (OS) of different groups. The log-rank test was used to compare the survival of different groups.

**Results:**

There were 41 patients who developed CIP, and the incidence rate of CIP was 18.5%. Univariate and multivariate logistic regression analyses showed that low pretreatment hemoglobin (HB) and albumin (ALB) levels were independent risk factors for CIP. Univariate analysis suggested that history of chest radiotherapy was related to the incidence of CIP. The median OS of the CIP group and non-CIP were 15.63 months and 30.50 months (HR:2.167; 95%CI: 1.355-3.463, *P*<0.05), respectively. Univariate and multivariate COX analyses suggested that a high neutrophil-to-lymphocyte ratio (NLR) level, a low ALB level and the development of CIP were independent prognostic factors for worse OS of advanced NSCLC patients treated with ICIs. Additionally, the early-onset and high-grade CIP were related to shorter OS in the subgroup.

**Conclusion:**

Lower pretreatment HB and ALB levels were independent risk factors for CIP. A high NLR level, a low ALB level and the development of CIP were independent risk factors for the prognosis of advanced NSCLC patients treated with ICIs.

## 1 Introduction

Lung cancer ranks first among all causes of cancer-related deaths around the world (1), while non-small cell lung cancer (NSCLC) accounts for more than 85% of lung cancers. Several clinical trials have confirmed that PD-1 inhibitors alone or combined with first-line chemotherapy for advanced NSCLC can bring significant survival benefits (2–4). However, the subsequent adverse reactions can not be ignored. Immune checkpoint inhibitors (ICIs) may cause immune-related adverse events(irAEs) such as rash, pruritus, pneumonitis, diarrhea, immune-mediated colitis, hepatitis and endocrine system problems (5–7). Among them, immune checkpoint inhibitor-related pneumonitis (CIP), which is a rare but fatal immune-related adverse reaction, has an incidence of 2% to 5%, with a mortality rate of 20% for grade 3 or higher CIP (8). The occurrence of CIP may also be associated with the tumor type, with a meta-analysis showing that, compared with patients suffering other cancers, patients with lung cancer are more likely to experience all-grade or high-grade CIP (9).

Previous studies have suggested that age, smoking history, preexisting lung diseases, history of chest radiotherapy, and the combination of two or more ICIs may be associated with the development of CIP (10–13). However, the sample size of CIP patients in these studies was small, and more influencing factors of CIP warrant further investigation. Hematologic inflammatory parameters can reflect the inflammatory status of the body; they have the advantages of being easily available, economical and convenient and play an important predictive role in the prognosis of tumors. The most explored parameters are the neutrophil-to-lymphocyte ratio (NLR) (14) and platelet-to-lymphocyte ratio (PLR) (15). Studies on the hematological inflammatory parameters of CIP are rarely reported. Thus, this study aimed to explore the risk factors for CIP, the relationship between hematological inflammatory parameters and the occurrence of CIP, and the survival of CIP patients.

## 2 Materials and methods

### 2.1 Study population

The included population was 222 advanced NSCLC patients treated with ICIs at the First Affiliated Hospital of Zhengzhou University between December 2017 and November 2021.

There were 145 patients who were treated with ICIs as first-line treatment, and 77 patients received immunotherapy as second- or further-line treatment. According to the ASCO guideline, CIP was diagnosed on the basis of computed tomography scans and clinical manifestations, excluding the diagnosis of disease progression, lung infection, and radiation pneumonitis (16). The treating investigators graded the severity of the pneumonitis using the Common Terminology Criteria for Adverse Events (CTCAE) version 5.0. The study was conducted following the guidelines of Declaration of Helsinki and approved by the Ethics Committee of the First Affiliated Hospital of Zhengzhou University (2022-KY-1316-001).

### 2.2 Data collection

Age, sex, smoking status, primary tumor type, clinical stage, underlying lung disease, whether targeted therapy was used, therapeutic regimen, hematological indexes within 1 week prior to immunotherapy, and history of prior radiotherapy were all obtained from medical records for all patients. The NLR was calculated as the neutrophil count/lymphocyte count. Baseline was defined as the moment to initiate ICIs; overall survival (OS) was defined as the interval between the start of immunotherapy and the date of death owing to any reason, or the last follow-up. We conducted the last follow-up up to June 29, 2022, by telephone and medical records.

### 2.3 Statistical analysis

All statistical analyses were conducted using SPSS version 21.0, and the results were then plotted by GraphPad prism version 8.0. The Kolmogorov‒Smirnov test was used to determine whether continuous data had a normal distribution. Continuous data with a normally distributed distribution are reported as the mean ± standard deviation, and were compared by Student’s *t* test. Categorical variables are summarized as the number of patients and percentages and were compared by the chi-square test or Fisher’s exact test. Logistic regression was used to evaluate risk factors of CIP, and the Kaplan‒Meier curve was used to describe the OS of different groups. The log-rank test was performed to compare the survival of different groups. The Cox regression method was used to evaluate the correlation of CIP and clinical characteristics with OS. *P*<0.05 was considered a statistically significant difference.

## 3 Results

### 3.1 Characteristics of CIP

According to whether they developed CIP or not before the end of follow-up, 222 patients were divided into a CIP group (n=41) and a non-CIP group (n=181). The numbers of patients with grade 1, 2, 3, 4 and 5 CIP were 10(24.39%),18(43.90%), 7 (17.07%), 6(14.63%) and 0 (0.00%) respectively. The median time from ICI initiation to the occurrence of CIP was 109 days [(interquartile range(IQR) :30-221] after ICI treatment, and the incidence of CIP was approximately 18.5%. The most common symptoms were cough (78.05%), fever (43.90%), dyspnea (53.66%) and chest tightness (34.15%). Early-onset CIP was defined as occurring within 6 weeks after commencement of ICI treatment, and late-onset CIP was defined as occurring beyond 6 weeks after starting ICI treatment (17). There were 12 patients who developed early-onset CIP, while 29 patients developed late-onset CIP. The results are shown in **Table 1**.

**Table 1:** Details of CIP.

Characteristics	Number
Grade of CIP	
1	10 (24.39%)
2	18 (43.90%)
3	7 (17.07%)
4	6 (14.63%)
5	0(0.00%)
Time from ICIs initiation to the occurrence of CIP(days)	109 (IQR: 30-221)
Occurrence within 6weeks	12 (29.27%)
Common symptoms	
Cough	32 (78.05%)
Fever	18 (43.90%)
Dyspnea	22 (53.66%)
Chest tightness	14 (34.15%)

### 3.2 The relationship between the occurrence of CIP in advanced NSCLC patients treated with ICIs and hematological parameters and clinical characteristics

The results of univariate analysis showed that there were no statistically significant differences between the two groups in terms of age, pathological type, preexisting lung disease, smoking status, immunotherapy combined with chemotherapy, brain metastases, the type of ICIs, NLR or monocytes (*P*>0.05). There was a difference in the history of chest radiotherapy between the two groups; 22.0% of the patients in the CIP group and 8.8% of the patients in the non-CIP group had a history of radiotherapy. The mean baseline hemoglobin (HB) level was lower in the CIP group (113.34 ± 15.20 g/L) than in the non-CIP group (124.37 ± 17.92 g/L) (*P*<0.001, *t*=3.653). The pretreatment albumin (ALB) level was lower in the CIP group (37.55g/L, IQR: 34.38-40.35) than in the non-CIP group (39.65g/L, IQR: 36.75-42.30, *P*<0.05) (**Table 2**).

**Table 2:** Basic information of patients in the two groups.

Variables	The occurrence of CIP			*P*
		YES (n=41)	NO (n=181)	
Age	<65	25 (61.0%)	115 (63.5%)	0.759
	≥65	16 (39.0%)	66 (36.5%)	
Gender	Male	28 (68.3%)	150 (82.9%)	0.034
	Female	13 (31.7%)	31 (17.1%)	
Smoking history	Yes	17 (41.5%)	93 (51.4%)	0.251
	Never	24 (58.5%)	88 (48.6%)	
Clinical Stage	IIIb-IIIc	16 (39.0%)	64 (35.4%)	0.659
	IV	25 (61.0%)	117 (64.6%)	
Preexisting Lung Disease	Yes	6 (14.6)	27 (14.9)	0.963
	No	35 (85.4)	154 (85.1)	
Histology	Squamous cell	18 (43.9%)	92 (50.8%)	0.537
	Adenocarcinoma	21 (51.2%)	76 (42.0%)	
	Others	2 (4.9%)	13 (7.2%)	
PD-L1 Expression	TPS≥50%	7 (17.1%)	26 (14.4%)	0.594
	1%≤TPS<50%	8 (19.5%)	50 (27.6%)	
	TPS<1%	10 (14.4%)	50 (27.6%)	
	Unknown	16 (39.0%)	55 (30.4%)	
Brain Metastases	Yes	8 (19.5%)	30 (16.6%)	0.652
	No	33 (80.5%)	151 (83.4%)	
Chest Radiotherapy History	Yes	9 (22.0%)	16 (8.8%)	0.034
	No	32 (78.0%)	165 (91.2%)	
ALK/EGFR-TKIs History	Yes	12 (29.3%)	26 (14.4%)	0.022
	No	29 (70.7%)	155 (85.6%)	
Therapy Protocols	Immune monotherapy	2 (4.9%)	20 (11.0%)	0.366
	ICIs± chemotherapy	39 (95.1%)	161 (89.0%)	
Treatment Line	1st line	24 (58.5%)	121 (66.9%)	0.313
	≥2nd line	17 (41.5%)	60 (33.1%)	
ICIS	PD-1	39 (95.1%)	177 (97.8%)	0.676
	PD-L1	2 (4.9%)	4 (2.2%)	
Laboratory Findings	ALB (g/L)	37.6 (34.4, 40.4)	39.7 (36.8, 42.3)	0.006
	HB (g/L)	113.3 ± 15.2	124.4 ± 17.9	<0.001
	NLR	4.0 (2.3,5.4)	3.0 (2.1,5.4)	0.329
	Monocyte (10^9/L)	0.6 (0.4,0.8)	0.6 (0.4,0.7)	0.824

### 3.3 Results of multivariate analysis

**Figure 1:**
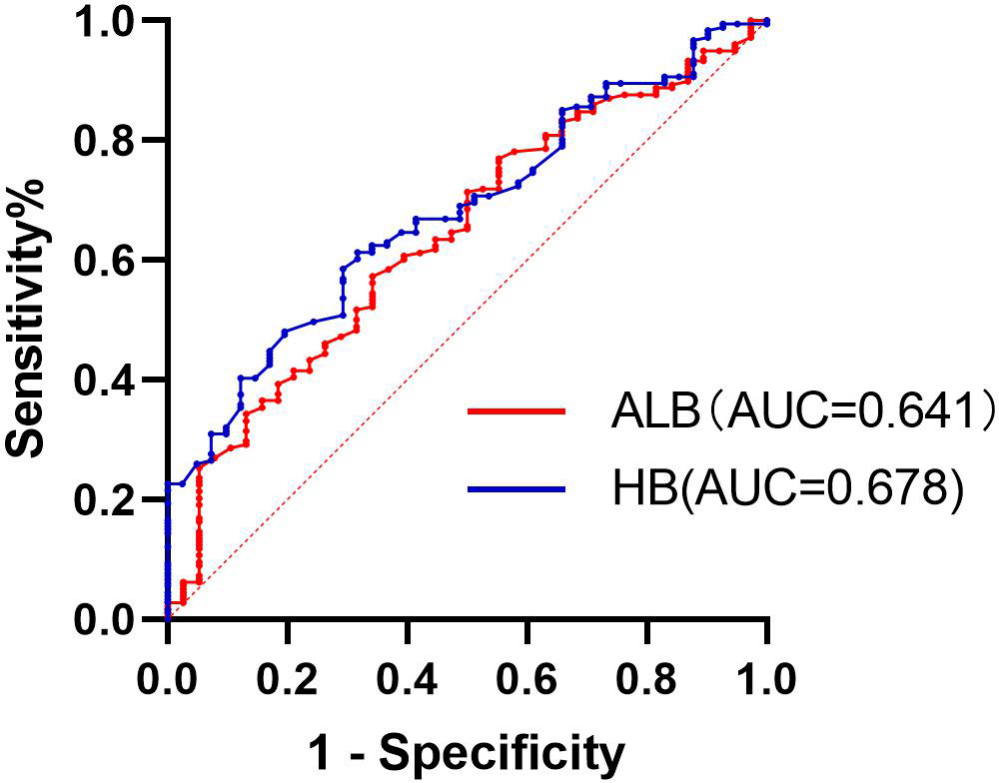
The ROC curve of ALB and HB for predicting the occurrence of CIP.

Multivariate logistic regression analysis showed that pretreatment HB and pretreatment ALB levels were independent predictive factors for CIP (**Table 3**). The best cutoff value was obtained by plotting the receiver operating characteristic (ROC) curve (**Figure 1**) with the occurrence of CIP before the end of follow-up as the status variable and pretreatment HB and ALB as the test variables. The area under the curve (AUC) of pretreatment HB value was 0.678 (95%CI: 0.596-0.759, *P*<0.001), with the highest predictive value at a pretreatment HB value of 120.9 g/L, resulting in a sensitivity of 68.3% and a specificity of 61.3% for predicting the occurrence of CIP. The AUC of pretreatment ALB value was 0.641 (95% CI:0.549-0.734, *P*<0.05), with the highest predictive value at a pretreatment ALB value of 38.75 g/L, resulting in a sensitivity of 65.8% and a specificity of 57.3% for predicting the occurrence of CIP (*P*<0.05; **Figure 1**).

**Table 3:** Analyses of risk factors for the occurrence of CIP.

Variables	Univariate analysis			Multivariate analysis		
	*P*	OR	95% CI	*P*	OR	95% CI
Male	0.038	0.445	0.208-0.955	0.112	0.481	0.195-1.186
EGFR/ALK-TKI history	0.025	2.467	1.119-5.349	0.085	2.293	0.893-5.889
Chest radiotherapy history	0.020	2.900	1.179-7.135	0.067	2.711	0.934-7.871
ICI treatment ≥2nd line	0.314	0.700	0.350-1.401			
Smoking history	0.253	1.492	0.751-2.964			
HB (g/L)	0.001	0.965	0.946-0.985	0.029	0.974	0.951-0.997
NLR	0.904	1.006	0.910-1.113			
ALB (g/L)	0.015	0.912	0.847-0.982	0.045	0.919	0.846-0.998

### 3.4 Prognosis

**Figure 2:**
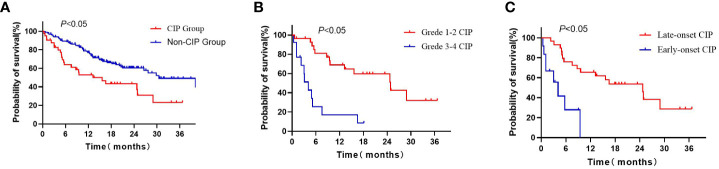
Kaplan–Meier curves for OS of advanced NSCLC patients: **(A)** Kaplan–Meier curves for OS of CIP patients and non-CIP patients; **(B)** Kaplan–Meier curves for OS of grade 1-2 CIP and grade 3-4 CIP patients; **(C)** Kaplan–Meier curves for OS of late-onset CIP and early-onset CIP patients. CIP, imune checkpoint inhibitor-related pneumonitis; OS, overall survival.

The median OS of the CIP group (15.63 months, 95% CI: 6.33-24.94) was shorter than that of the non-CIP group (30.50 months, 95% CI: 21.67-39.33), and there was a statistically significant difference (*P*< 0.05; **Figure 2A**). The median OS of the grade 3-4 CIP group (4.07months, 95%CI:2.32-5.82) was shorter than that of the grade 1-2 CIP group (24.87months, 95%CI:11.66-38.08), and there was a statistically significant difference (*P*<0.05, **Figure 2B**). The median OS of early-onset CIP patients (4.07 months, 95% CI: 1.57-6.56) was shorter than that of the late-onset CIP patients (24.73months, 95% CI: 14.66-34.81, *P*<0.05, **Figure 2C**). The Cox multivariate regression analysis results showed that a lower pretreatment ALB level, the occurrence of CIP and a high baseline NLR value were negative predictors for the OS of NSCLC patients treated with ICIs (**Table 4**).

**Table 4:** Analyses of factors potentially associated with overall survival of the advanced NSCLC patients treated with ICIs.

Variables	Univariate analysis			Multivariate analysis		
	*P*	HR	95% CI	*P*	HR	95% CI
Male	0.617	1.147	0.671-1.960			
Age(y)	0.047	1.026	1.000-1.052	0.678	1.005	0.980-1.031
Smoking history	0.139	0.729	0.480-1.107			
Preexisting lung disease	0.448	1.257	0.697-2.265			
Chest radiotherapy history	0.412	0.759	0.392-1.467			
ICI treatment ≥2nd line	0.528	0.871	0.568-1.337			
Occurrence of CIP	0.001	2.167	1.355-3.463	0.006	2.019	1.219-3.345
NLR	0.001	1.083	1.032-1.137	0.009	1.080	1.019-1.145
ALB (g/L)	<0.001	0.909	0.870-0.950	0.011	0.943	0.901-0.987
HB (g/L)	<0.001	0.976	0.965-0.987	0.093	0.988	0.975-1.002

## 4 Discussion

At present, there are still a few reports about CIP, some from clinical trials and some from the real-world, but there are no clear conclusions about its incidence and risk factors. Some clinical trials and reports have shown that the incidence of CIP is approximately 5% (18, 19). A multi-institutional cohort study recently has found that the risk of pneumonitis associated with PD-1/PD-L1 inhibitors compared with non-immunotherapy was 2.49% (95% CI: 1.50%-3.47%), and the median time to the onset of CIP was 3.9 months (IQR: 2.1-7.3) (20). In our study, the median time to the occurrence of CIP was 109 days (IQR: 30-221) after ICI treatment, and the incidence of CIP was approximately 18.5%. Some reports have shown that the incidence of CIP is higher than in those clinical trials (21, 22), which is consistent with our results. The rising occurrence of CIP in the real world may be due to the increased vigilance of clinicians toward CIP in recent years. The incidence rate of CIP requires more real-world data for feedback and verification in studies with larger sample size.

Our study demonstrated that the occurrence of CIP was increased in patients who had undergone thoracic radiotherapy. Although the results of our multivariate logistic regression analysis showed that previous chest radiotherapy was not an independent risk factor for CIP, the Keynote-001 trial demonstrated that patients who received thoracic radiotherapy before pembrolizumab were more likely to develop CIP of any grade than those who did not (23). This may be due to the damage to lung function caused by a certain dose of radiation to the lung, the continuous low-level release of inflammatory factors caused by radiotherapy, and ICIs promoting an increase in the level of inflammatory factors. This also suggests that radiation has an immunomodulatory effect. Radiation-induced cell death generates molecular signals and inflammatory cytokines that facilitate the ability of dendritic cells to deliver antigens to T cells (24). Therefore, radiotherapy is often used in combination with ICIs for NSCLC because of their synergistic effects, but we should be wary of the increase in toxicity during application.

One study found that a low serum ALB level with pembrolizumab was an independent predictor of CIP (25), consistent with the finding of our study. In addition, Hu et al. found that increased ALB concentration was associated with improved lung function (26). ALB is an acute phase reactant that can show the inflammatory state of the body; a decrease in ALB may be related to the inflammatory state of the body, and these mechanisms lead to the occurrence of CIP. In addition, to our knowledge, we are the first to find that a lower pretreatment HB level is associated with the occurrence of CIP. Although no study has reported that HB values can predict the occurrence of CIP, He et al. found that low HB was independently associated with the occurrence of community-acquired pneumonia in pregnant women (27). HB plays the role of transporting oxygen, and its deficiency is related to hypoxia, which may promote the deficiency of lung function, making patients susceptible to pneumonitis. On the other hand, decreased HB levels are related to weakened immunity (28), leading to insufficient cellular immunity, which also promotes the development of pneumonitis to some extent. Zhao et al. found that anemia was also correlated with T-cell deficiency in mice (29), so the decline in HB may also predispose people to CIP through immunosuppression. As we know, previous anticancer treatment can have an impact on HB and ALB levels. Bone marrow suppression induced by chemotherapy or radiotherapy can make HB decrease, and gastrointestinal adverse effects cause patients to lack appetite, malnutrition, and ALB decline. In our study, there was no difference in HB and ALB levels between patients treated with ICIs in the first-line and second-line and beyond (**Supplementary Table 1**). In addition, we found that the treatment lines of immunotherapy did not correlate with CIP. However, a different result has been reported. Khunger et al. have conducted a Meta-analysis showing that the incidence of all grades of CIP was significantly higher in treatment naive patients than in previously treated ones (30). Whether the treatment lines of immunotherapy are related to the incidence of CIP by affecting levels of HB and ALB still needs to be explored in the future.

In this study, the OS of patients in the CIP group was shorter than that of patients in the non-CIP group. We also found that patients who developed early-onset and high-grade CIP had shorter OS than those who developed late-onset and low-grade CIP. Previous research showed that compared with patients without irAEs, the OS of patients with irAEs was substantially prolonged (31), suggesting that the presence of irAEs may be related to prognosis. A recent study by Haratani et al. showed that the occurrence of any irAE was related to longer PFS and OS in advanced NSCLC patients (10), and other studies have reported similar results (17, 31–33). In light of these findings, irAEs are generally regarded as indicators of NSCLC patients’ improved response to PD-1 inhibitors and longer survival. Nevertheless, the number of patients with CIP included in these reports was quite small, and some studies reported different findings. One study showed that grade 1-2 CIP was linked to good OS; however, grade 3-4 CIP was not (34). Fukihara et al. revealed that CIP patients had considerably shorter OS than non-CIP patients (25). This may be in part because patients with CIP frequently need to stop using PD-1 inhibitors since they can induce deadly respiratory failure, unlike those with skin responses or thyroid problems. What's more, CIP directly affects the patient’s respiratory function and thus survival. In addition, some studies have shown that the use of glucocorticoids may shorten patient’s OS (35, 36), which a proportion of CIP patients usually have difficulty avoiding using. These factors may together contribute to the shortened survival of CIP patients.

In addition, this study found that pretreatment ALB was associated with OS in patients with advanced NSCLC receiving immunotherapy. ALB reflects nutritional status and response to inflammation and is related to the treatment outcome of NSCLC. Hypoalbuminemia has been reported to be associated with low survival rates in tumor patients (37). Our study also found that a high NLR before treatment was associated with a worse prognosis after immunotherapy. The NLR is an effective index to reflect the degree of the inflammatory response and immune status. The systemic inflammatory response is considered to be closely related to the occurrence and progression of tumors. Some studies have shown that high levels of the NLR are closely related to the poor prognosis of lung cancer (38), which was in agreement with our results.

The findings of this study on CIP are valuable for clinicians to better understand the risk factors and prognosis for the development of CIP, and help us to recognize populations with these characteristics. In this way, we can take full account of possible toxicity risks when immunotherapy is administered to these patients, and be alert to the incidence of CIP and make appropriate clinical decisions to obtain the maximum benefit of immunotherapy. Compared to the current published studies facing the same topic, our work had several strengths: First, we were the first to find that a lower pretreatment HB level was associated with the occurrence of CIP. In addition, we have found that patients who developed CIP had a worse prognosis than those who did not; Third, we found that early-onset and high-grade CIP were associated with a worse prognosis than late-onset and low-grade ones. However, there were two limitations in our study: First, it was a retrospective study, and we could not determine the patient’s treatment strategy. In addition, there was no preassessment of the patient’s lung function, and the sample size was not large enough. Treatment modalities still need to be explored in studies with larger sample sizes.

## 5 Conclusion

Lower pretreatment HB and ALB levels were independent predictors of CIP. The occurrence of CIP, a lower pretreatment ALB level, and a high pretreatment NLR value were negative predictors for the prognosis of NSCLC patients treated with ICIs.

## Data availability statement

The raw data supporting the conclusions of this article will be made available by the authors, without undue reservation.

## Ethics statement

The study design was reviewed and approved by the Ethics Committee of the First Affiliated Hospital of Zhengzhou University. Written informed consent for participation was not required for this study in accordance with the national legislation and the institutional requirements.

## Author contributions

XL: study design, data collection and analysis, writing-original draft, and writing – review & editing. NH: study design, data collection, and writing – review & editing. SY: study design, data collection, and writing – review & editing. JL: study design, data collection, and writing– review & editing. LW: formal analysis and writing – review & editing. All authors contributed to the article and approved the submitted version.
