# Supplementary material for: Predictive factors and prognosis of immune checkpoint inhibitor-related pneumonitis in non-small cell lung cancer patients
Source: Front Oncol. 2023 Apr 26;13:1145143. doi: 10.3389/fonc.2023.1145143 (PMC10169751; doi:10.3389/fonc.2023.1145143)
Supplement: Supplementary file 1 [file Table_1.docx]

| Variables | First-line treatment | Second- or higher-line treatment | *Z* | *P* |
| --- | --- | --- | --- | --- |
| Pretreatment hemoglobin | 125.0(111.8,137.0) | 123.0(108.0,130.9) | -1.909 | 0.056 |
| Pretreatment albumin | 39.2(36.3,41.8) | 39.1(36.2,42.2) | -0.491 | 0.623 |

Table 1 Comparison of treatment lines and hemoglobin and albumin
